# Design, construction, and technical implementation of a web-based interdisciplinary symptom evaluation (WISE) - a heuristic proposal for orofacial pain and temporomandibular disorders

**DOI:** 10.1186/s10194-016-0670-5

**Published:** 2016-08-31

**Authors:** Dominik A. Ettlin, Isabelle Sommer, Ben Brönnimann, Sergio Maffioletti, Jörg Scheidt, Mei-Yin Hou, Nenad Lukic, Beat Steiger

**Affiliations:** 1 Orofacial Pain Unit of the Center of Dental Medicine, University of Zurich, Zurich, Switzerland; 2 S3IT: Service and Support for ScienceIT, University of Zurich, Zurich, Switzerland; 3 Institut für Informationssysteme, Hochschule für Angewandte Wissenschaften Hof, Hof, Germany

**Keywords:** Personalized medicine, Patient-reported outcome measures, Orofacial pain, Temporomandibular disorders, Questionnaire

## Abstract

**Background:**

Medical symptoms independent of body location burden individuals to varying degrees and may require care by more than one expert. Various paper and computer-based tools exist that aim to comprehensively capture data for optimal clinical management and research.

**Methods:**

A web-based interdisciplinary symptom evaluation (WISE) was newly designed, constructed, and technically implemented. For worldwide applicability and to avoid copyright infringements, open source software tools and free validated questionnaires available in multiple languages were used. Highly secure data storage limits access strictly to those who use the tool for collecting, storing, and evaluating their data. Concept and implementation is illustrated by a WISE sample tailored for the requirements of a single center in Switzerland providing interdisciplinary care to orofacial pain and temporomandibular disorder patients.

**Results:**

By combining a symptom- burden checklist with in-depth questionnaires serving as case-finding instruments, an algorithm was developed that assists in clarifying case complexity and need for targeted expert evaluation. This novel modular approach provides a personalized, response-tailored instrument for the time- and cost-effective collection of symptom-burden focused quantitative data. The tool includes body drawing options and instructional videos. It is applicable for biopsychosocial evaluation in a variety of clinical settings and offers direct feedback by a case report summary.

**Conclusions:**

In clinical practice, the new instrument assists in clarifying case complexity and referral need, based on symptom burden and response –tailored case finding. It provides single-case summary reports from a biopsychosocial perspective and includes graphical symptom maps. Secure, centrally stored data collection of anonymous data is possible. The tool enables personalized medicine, facilitates interprofessional education and collaboration, and allows for multicenter patient-reported outcomes research.

## Background

Primary, secondary and tertiary care providers may all be involved in the diagnosis and management of symptoms related to tissue dysfunction and pain disorders. Gathering valid and reliable data in all types of clinical setting is essential for high-quality personalized care and research [1]. Patient self-report data are increasingly recognized as a valuable resource for this purpose. Yet, in the context of patient consultations, it is often difficult to systematically and prospectively collect high-quality data. This aspect is further complicated in studies that aim to comprehensively assess physical and psychosocial parameters, e.g., functionality, pain interference, beliefs and expectations; pain catastrophizing, social roles, functioning, and interactions; emotional distress, and sleep.

The seminal 1992 publication of the Research Diagnostic Criteria for Temporomandibular Disorders (RDC/TMD) and the subsequent refinement in the form of Diagnostic Criteria for Temporomandibular Disorders (DC/TMD) in 2014 represented a paradigm shift in the evaluation and diagnosis of patients with TMD, a heterogeneous group of disorders affecting the jaw joint and surrounding tissues [2, 3]. The most salient novel feature contrasting with previous TMD diagnostic systems was the introduction of the biopsychosocial model into dental medicine. This concept includes not only the assessment of somatic signs and symptoms (Axis I) but also the biobehavioral domain (Axis II). The screening of patients for psychosocial burdens aimed to appropriately refer patients for expert assessment and interventions to address non-somatic barriers to TMD recovery. Subsequent prospective cohort studies supported the clinical utility of Axis II instruments [4–6]. Psychosocial factors have the potential to affect treatment responses not only of OFP and TMD sufferers, but of many types of chronic pain [7–9]. Management of diverse biopsychosocial issues and/or comorbidities is relevant for some — but not all — individuals experiencing OFP and TMD. Thus, individuals seeking health care vary greatly in their subjective complaints, personal histories, and comorbid conditions. The diverse clinical symptoms of identical pathologies may be attributable to differing environmental, psychosocial, and genetic factors. Common approaches for profiling patients and phenotyping disorders may include the following tools: checklists, questionnaires, interviews, physical examinations, imaging, laboratory tests, and psychological/psychiatric evaluations.

The administration of a barrage of measures can significantly increase patient burden and decrease compliance. Recently, data collection methods that are adapted to a patient’s unique history (rather than a lengthy survey including questions that would not apply to them) have become popular [10, 11]. For this purpose, a checklist can call attention to a symptom and ensure that nothing of importance is overlooked. Since multiple somatic and psychological symptoms frequently coexist in OFP and TMD patients, case-finding instruments have been developed for initiating diagnostic procedures that facilitate optimized treatment. For this purpose, cost- and time-effective questionnaires validated in the primary care setting exist that are capable to clarify the need for expert evaluation of individuals possibly suffering from migraine, tinnitus, anxiety, depression, sleep disorders, etc. A possible way to build a tool for comprehensive patient assessment is to combine a symptom-burden oriented checklist with various in-depth questionnaires serving as case-finding instruments.

Traditionally, data collected via paper-based questionnaires can be used for clinical care and research. However, it is time-consuming and costly to extract their data. Paper-based questionnaires are often disliked by patients, data are sometimes unreadable, and missing items are challenging for statistical data analysis [12]. Among others, the US Institute of Medicine advocates using information technology to support patient-centered care and evidence-based decisions [13, 14]. Electronic healthcare records are increasingly implemented in many countries. E.g., in the U.S., a Medicare and Medicaid Electronic Health Care Record Incentive Program was recently established to encourage widespread adoption of an electronic health record [15]. Capturing patient reported information electronically results in a more accurate and complete data set, improved protocol compliance, avoidance of secondary data entry errors, easier implementation of skip patterns, less administrative burden, high respondent acceptance, reduced sample size requirements, and potential cost savings. The increasing number of patients who are already familiar and comfortable with electronic devices are likely to prefer this format of delivery [16]. Health information technology (health IT) enables the collection of large amounts of patient care data in electronic form, but this requires new architectures, techniques, algorithms, and analytics for data management and for extracting knowledge [17]. In the U.S., a collaborative health outcomes information registry (CHOIR) is currently being built from patient data provided by US American care centers [18]. The structure and composition of its questions addressing OFP and TMD, its algorithm, and its scoring system are, however, not publicly available.

The goal of this project was to design, construct, and implement a modular, universally accessible, web-based instrument for interdisciplinary symptom evaluation (WISE) for subject-tailored assessment of OFP and TMD prior to clinical interviews. We aimed to clarify symptom-burden by a checklist and with case-finding instruments in the form of publicly available, in-depth questionnaires to create response-tailored assessments. Copyright issues were avoided and a highly secure data storage location free of third party interests was selected.

## Methods

### Open source software tools

#### LimeSurvey™ (LS)

To design and construct the WISE for OFP and TMD, we used LimeSurvey™ 2.05+ (150310), which is a platform-independent open source framework for the development of internet based surveys [19]. LS runs on any web server and the data is stored in a MySql database. To ensure privacy, data can be transmitted via secure https. As a survey framework, LS contains predefined response formats including single and multiple choice, array responses, and equations. Equations enable calculation of scores that serve as filters to stratify for additional in-depth questions. LS implements the construction of multilingual surveys. Data can be exported in different formats, such as SPSS, Excel, and others.

#### ImageMapster (IM)

IM is a JQuery-based tool for using image maps for data entry [20]. Predefined areas of a background image can be selected to graphically represent pain on a body schematic. IM can easily be included in the LS question code. In the WISE, IM was used for the assessment of OFP location and severity.

### Data security and storage

**Fig. 1:**
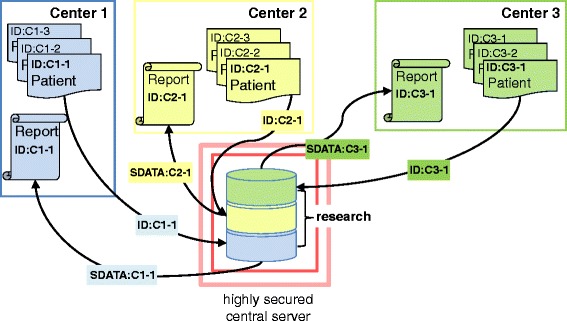
Overview of data exchange options between multiple patient management centers and a secure central data collection server. For clinical practice, customized single case reports available only to the supplying clinic are generated from centrally stored data that are linked by a unique identification number (ID). For research purposes, anonymized data clusters can be merged, thus enabling multicenter research projects

Maintaining participant privacy is critical. The WISE can run on any highly secure webserver. Data security and storage can be managed in a single country or on widespread servers. For optimal data security, data linked to patient identity are stored independent of survey data. The latter are stored in anonymized form on a highly secure central server, whereas patient identity data are stored locally in clinics or centers. Single-case summary reports are generated by linking local and central data via a unique identification number (ID). In this process, the only data exchanged is an anonymous ID and anonymized survey data (SDATA). During initial data collection, the authors managing patients at the Orofacial Pain Unit of the Center of Dental Medicine, University of Zurich (UZH) established a central secure host at Service and Support for Science IT of the UZH, adhering to Swiss federal and cantonal laws for privacy protection. Figure 1 illustrates the current setup at the University of Zurich, Switzerland.

### WISE concept, structure and content

The WISE was designed to assist clinical decision making. A key requirement was the ability to capture symptoms that are commonly experienced by patients reporting to a given health care provider setting. In order to illustrate the pragmatic implementation of this technical tool and its underlying concept, we present a WISE sample structure tailored for the requirements of the interdisciplinary orofacial pain unit at the University of Zurich, Switzerland, where patients seek care for a broad variety of musculoskeletal, neuropathic and idiopathic orofacial pains conditions. From a technical-methodological perspective, adapting the software to satisfy various content requirements of similar or other settings is easily accomplished owing to the tool’s modular and modifiable design.

Conceptually, the WISE was structured to assess patients in three stages: 1) assessment of symptom burden by a checklist, 2) response-tailored in-depth analyses of burdening symptoms by case-finding validated questionnaires, and 3) targeted expert evaluation(s) of burdening symptoms identified as likely being part of a defined condition.

Importantly, the term “symptom” relates to the subjective experience of an unusual state (abnormal function or feeling) that is not directly measurable. Making perceptual decisions and classifying bodily sensations as possibly harmful is an inherent part of interoception which influences the subjective experience of symptom-related burden [21]. E.g. a headache may be painful, but perhaps not sufficiently burdening the person to take medication. Analogously, a jaw joint sound due to an anterior disc displacement with reduction may be slightly annoying to some people, yet highly burdening to others. Uncertainty about the harmfulness of bodily sensations typically influences the individually perceived symptom burden [22]. It is for these reasons that the WISE checklist focuses on degree of somatic and psychological symptom burdens. Whether burdening symptoms are part of an expert defined condition can be further evaluated by in-depth questionnaires which serve as validated case-finding instruments for clarification. Clinicians are thereby alerted about the possible indication for further interdisciplinary expert evaluation The latter may involve more refined validated instruments such as e.g. a structured clinical interview and/or validated clinic assessment for establishing a diagnosis according to DC/TMD, International Classification of Headache Disorders, Diagnostic and Statistical Manual of Mental Disorders, etc.

#### Questionnaires by which checklist content was thematically aligned

Co-existence of multiple somatic and psychological symptoms is prevalent in OFP and TMD patients. For heuristic purposes, the item content of the WISE symptom burden checklist was thematically aligned with questionnaires commonly addressing these diverse symptom domains. Notably, the adaptation of items and/or of entire questionnaires resulted in the loss of their originally validated psychometric properties. The symptom burden checklist items were taken as is or adapted from the following instruments.

##### DC-TMD Symptom Questionnaire (DC-TMD-SQ)

The DC-TMD-SQ has 14 items and is part of the DC/TMD algorithms, the validity of which is presented in Schiffman et al. [3]. It inquires about the presence of common symptoms associated with OFP and TMD.

##### Patient Health Questionnaire 15 (PHQ-15)

The PHQ-15 evaluates the severity of somatic symptoms [23]. It was never intended to diagnose a specific clinical entity.

##### PHQ-Stress

PHQ-Stress is a 10-item subscale of the Primary Care Evaluation of Mental Disorders (PRIME-MD) Patient Health Questionnaire that addresses burden by psychosocial stress [24].

#### Validated case-finding questionnaires

The following questionnaires clarify whether burdening symptoms are part of an expert defined condition and thus assist clinicians in identifying patients that might benefit from further targeted expert evaluation. Information on their coefficient alpha and test-retest reliability has been included when published.

##### Patient Health Questionnaire 4 (PHQ-4)

The PHQ-4 screens for anxiety and depression in primary care patients [25, 26]. It consists of two subscales GAD-2 (item 1 and 2 of the General Anxiety Disorder Questionnaire 7) and PHQ-2 (item 1 and 2 of the Patient Health Questionnaire 9). Items are scored on a four-point ordinal rating scale. Scores can be calculated for the two subscales (maximum score = 6) as well as overall (maximum score = 12). Total scores of 6 to 8 or subscale scores of 3 to 4, respectively, indicate a *possible* disorder (“yellow flag”). Total scores above 8 or subscale scores above 4, respectively, are suggestive of a *probable* disorder (“red flag”). The following coefficient alpha have been reported: PHQ-4: 0.87; PHQ-2: 0.75; GAD-2: 0.82 [25], and for test-retest reliability: PHQ-4: 0.81; PHQ-2: 0.77; GAD-2: 0.69 [27].

##### General Anxiety Disorder Screener 7(GAD-7)

The GAD-7 assesses general anxiety in primary care patients [28, 29]. High correlations with disability measures were found [30]. Seven items covering different aspects of general anxiety are scored (using the same scale as the PHQ-4). Summary scores range from 0 to 21 and indicate anxiety levels of “none/minimal” (0–4), “mild” (5–9), “moderate” (10–14), or “severe” (>14). The following coefficient alpha has been reported: 0.92 and fortest-retest reliability: 0.83 [31].

##### Patient Health Questionnaire 9 (PHQ-9)

The PHQ-9 assesses severity of depression [32]. Like the GAD-7, it correlates with functional impairment [30]. Nine items covering different aspects of depression are scored (using the same scale as the PHQ-4). Summary scores range from 0 to 27, indicating depression levels of “none/minimal” (0–4), “mild” (5–9), “moderate” (10–14), “moderately severe” (15–19), or “severe” (> 19). A cut-off score range of 8–11 has been recommended for expert evaluation referral [31, 33]. The following coefficient alpha has been reported: 0.89 and for test-retest reliability: 0.84.

##### PHQ-Stress

The cut-off scores are 10 for “medium” and >14 for “severe” burden by psychosocial stress [24]. No coefficient alpha nor test-retest reliability were reported.

##### Graded Chronic Pain Scale (GCPS) v2

The GCPS was originally developed for general population surveys and primary health care to improve prognostic categorization and treatment decisions [34]. Its prognostic validity in OFP and TMD research has been supported: Higher GCPS ratings are a risk factor for pain chronicity [4]. For clinical decision-making, matching TMD pain-related disability levels with appropriate treatment has been recommended [35]. GCPS consists of seven items measuring pain intensity and related disability, which were scored independently. The maximum disability score is six. Scores of 3–4 are interpreted as moderate impairment and 5–6 as severe impairment. The 90 days version of the scale was implemented for the WISE. The following coefficient alpha has been reported: 0.71 for TMD pain, test-retest reliability has not been reported.

##### Pain Catastrophizing Scale (PCS)

The PCS assesses catastrophizing thoughts and corresponding behavior [36]. Its 13 items were rated on a 5-point ordinal rating scale. The maximum score is 52, with a cut-off score of 30 [36]. The following coefficient alpha has been reported: 0.87 and for test-retest reliability: 0.75.

##### Insomnia Severity Index (ISI)

The ISI screens for sleep disorders by measuring the severity of insomnia problems, sleep-related satisfaction, and interference. Items were rated on a 5-point ordinal rating scale. The maximum score is 28, with a cut-off score of 14 [37–40]. The following coefficient alpha has been reported: 0.74, test-retest reliability was not reported.

##### Brief Illness Perception Questionnaire (B-IPQ)

The B-IPQ assesses cognitive and emotional representations of illness and health threat [41, 42]. Eight questions covering different aspects of illness perception were rated on a numeric rating scale ranging from 0 to 10. No cut-off score has been reported. Test-retest reliability of the single items of the B-IPQ range from .48 to .70, a coefficient alpha value was not reported.

##### Injustice Experience Questionnaire (IEQ)

The IEQ assesses injustice experienced due to accidents, injuries, or maltreatment [43]. Twelve items, which reflect the frequency of thoughts, beliefs, and emotions associated with injury, were rated on an ordinal scale ranging from 0 to 4. The maximum score is 48, with a cut off score of 30 representing a clinically relevant level of perceived injustice [30]. The following coefficient alpha has been reported: 0.92 and for test-retest reliability: 0.90.

##### Dysmorphic Concern Questionnaire (DCQ)

The DCQ assesses excessive preoccupation with imagined or actual, minimal defects in appearance that significantly influence psychosocial functioning [44, 45]. It consists of seven items, rated on an ordinal scale ranging from 0 to 3. The maximum score is 21 with cut off score of 9 suggesting a possible body dysmorphic disorder [44]. The following coefficient alpha for the DCQ has been reported: 0.85, test-retest reliability was not reported.

##### Tinnitus Handicap Inventory (THI-12)

The 12-item THI assesses tinnitus severity and its impact on daily life [46]. The summary score ranges from 0 to 24 indicating following levels of impairment by tinnitus: 0–6 denotes “*no handicap*”, 7–10 “*mild handicap*”, 11–14 “*moderate handicap*”, and >14 “*severe handicap*”. The following coefficient alpha has been reported: 0.88 and for test-retest reliability: 0.89.

##### Identification of Migraine (ID-Migraine™) screener

The three-item ID-Migraine™ screens for migraine headache [47, 48]. An affirmative response to two of three items discriminates migraine from other headaches. No coefficient alpha or test-retest reliability was reported for this instrument.

#### Other symptom exploration instruments

##### Jaw Function Questionnaire (JFQ)

The JFQ is a checklist of 12 daily jaw activities for assessing OFP and TMD related disability [49]. It was part of an earlier version of instruments to assess axis-II disorders of the RDC/TMD consortium. It was preferred over the Jaw Function Limitation Scale because the latter contains items unsuitable for vegetarians/vegans, leading to cultural bias. The answer options were expanded to “*no*” (=0), “*a little*” (=1), and “*a lot*” (=2) to better assess the impact of limitations. No diagnostic cut-off value applies. We added two measures for quantitative evaluation: 1) “jaw function” on a scale ranging from “*normal function*” (0) to “*no movement possible*” (10) and 2) “dietary restrictions” on a scale ranging from “*no restrictions*” (0) to “*liquid only*” (10) [50].

##### Somatosensory symptom checklist (SSC)

Somatosensory facial alterations are not systematically captured in orofacial pain questionnaires. For the WISE for OFPand TMD, we integrated an eight-item checklist that is used to assess posttraumatic neurosensory deficits or altered function [51]. No cut-off value applies.

### Administration

After receiving a referral email or letter, the clinic administrator registers the patient in a database. The login information for the WISE is sent to the patient by letter. Upon opening the survey, patients receive information about its purpose, duration, and privacy protection. Instructions, including short video-clips, are available for assistance. Upon submitting the questionnaire, a patient will be contacted by the clinic administration in order to schedule an appointment. This is the way that our clinic uses the WISE, but any different implementation is possible.

## Results

Similar to a patient chart, the WISE captures information according to the following structure:

1. General information/patient characteristics (gender, age, height, weight, known allergies, social and parafunctional habits, primary care and referring clinician, occupational status);
2. Chief complaint(s) and modulating factors;
3. Symptom burden checklist and related in-depth questionnaires;
4. Previous diagnoses and effects of prior treatments;
5. Privacy policy, informed consent.

### General structure and scoring of the symptom burden checklist

The checklist begins with items addressing symptom burden in various extra- and intraoral locations. Additional checklist items were included for comprehensive orofacial symptom assessment such as xerostomia, halitosis, dysphagia, tooth/jaw related dysmorphophobia, and obstructive sleep apnea. Pain-related questions were grouped by body areas. Checklist-item scoring was structured according to the PHQ-15 by grading the symptom-related burden.

### Thresholds for presenting case-finding tools and in-depth questions

Thresholds for presenting case-finding tools and in-depth questions can be adjusted, depending on particular clinic or research focus. Here, we suggest a low threshold for pain-related checklist items and a higher threshold for others. I.e., patients who check being bothered at least ‘a little’ for one or more pain items were offered additional in-depth questions which focus on capturing graphically pain location and intensity in the following areas: head/face, torso, and elsewhere on the body. Further questions address pain quality, onset, duration, time pattern, diurnal course, and pain-related disability (Table 1 and section Pain severity and location). The threshold is set to ‘bothered a lot’ for the presentation of the following case-finding tools: PHQ-9, GAD-7, IPQ, PCS, DCQ, IEQ, PHQ-S, THI-12, ISI.

**Table 1:** WISE items and checklist content sources, case-finding tools and other symptom exploration instruments

Symptom domain	Checklist item	Source	Threshold	In-depth assessment
Head and orofacial area	1) Toothache/oral pain (e.g., tongue, gums)	none		
	2) Pain/tightness in the jaw or face	DC-TMD-SQ:1		
	3) Ear pain, ear pressure, tinnitus (e.g., ringing noise)	DC-TMD-SQ:1	a little	THI-12
	4) Headache	DC-TMD-SQ:2	a little	ID-Migraine Screener
	Σ(1..4)		1	PAIN-head/face GCPS-head/face
	5) Limitation/pain upon mouth opening or closing (e.g. yawning)	DC-TMD-SQ: 9 & 13	a lot	modified JFQ
	6) Limitation/pain upon biting/chewing/talking/drinking	DC-TMD-SQ: 4 & 7	a lot	modified JFQ
	7) Temporomandibular joint (TMJ) noises (e.g., clicking, crepitus)	DC-TMD-SQ:8	a little	clicking crepitus other
	8) Tooth/jaw position (e.g., bite is incorrect)/physical appearance	none	a lot	DCQ
	9) Abnormal sensations in the mouth, lips or face that have negative effects (e.g., uncontrolled drooling)	SSC	a lot	SSC, modified JFQ
	10) Dry mouth/malodor/swallowing difficulties	none		
Other pain	11) Pain in the neck/shoulder	none		
	12) Pain in the back area	PHQ-15:2		
	13) Pain in the chest/abdomen/genitals	PHQ-15:1,4,6 & 11	a little	PAIN-torso
	14) Pain in the arms, legs	PHQ-15:3		
	Σ(11,12,14)		1	GCPS-B PAIN-body
Other symptoms	15) Worries about my chief complaint(s)	PHQ-S:1	a lot	B-IPQ, PCS
	16) Increased fatigue/loss of energy/unintentional weight loss or gain	PHQ-15:14	a lot	PHQ-9
	17) Snoring/apnea during sleep	none		
	18) Dizziness/nausea/fainting spells/shortness of breath/feeling your heart pound or race/indigestion	PHQ-15:7–10,13	a lot	PHQ-S
	19) Lack of time/work related stress/caring responsibilities/finances	PHQ-S:5–7	a lot	PHQ-S
	20) Lack of support/interpersonal conflicts/loneliness	PHQ-S:4,8	a lot	PHQ-S
	21) Different opinions of different caregivers/not been taken seriously	none	a lot	IEQ
	22) Stressful life events (something bad that happened recently or in the past with corresponding thoughts/dreams/feelings)	PHQ-S:9,10	a lot	PHQ-S

### Assembly of the WISE items (see Table 1 and 2)

#### DC-TMD Symptom Questionnaire (DC-TMD-SQ)

The DC-TMD-SQ consists of checklist items (items 1, 5, 8, 9, 13) and related in-depth sub items (items 2, 3, 4, 6, 7, 10, 11, 14).

**Table 2:** PHQ-4 screening items and thresholds for related case-finding tools. A value of >2 (yellow flag) was chosen for a further evaluation by GAD-7 and by PHQ-9. For item 27, a value of >1 was used as threshold for presenting the ISI

Checklist item (continued)	Source	Threshold	In-depth assessment
23) Feeling nervous, anxious or on edge	GAD-2	Σ > 2	GAD-7
24) Not being able to stop or control worrying			
25) Little interest or pleasure in doing things	PHQ-2	Σ > 2	PHQ-9
26) Feeling down, depressed, or hopeless			
27) Trouble falling or staying asleep, or sleeping too much	PHQ-9	> 1	ISI

Content of DC-TMD-SQ item 1 (pain in the jaw, temple, in the ear, or in front of the ear) was split into checklist items 2 and 3. Content of DC-TMD-SQ items 5 (headache) and 8 (joint noises) were added unaltered to the checklist. Content of DC-TMD-SQ item 9 (closed locking of the jaw) and item 13 (open locking of the jaw) were grouped into checklist item 5. The recall period for symptom presence was limited to 30 days, consistent with other checklist items.

The content of DC-TMD-SQ items 2, 3, and 6 were placed in the in-depth pain exploration section (see section Pain duration and Time pattern). Content of DC-TMD-SQ sub items 4 and 7 were covered in the JFQ, which focuses on disabling rather than aggravating aspects of OFP and TMD. Finally, content of DC-TMD-SQ sub items 10, 11 and 14 (corresponding to DC-TMD-SQ checklist items 9 and 13) were omitted because these aspects were considered better explored in the clinical interview.

#### Patient Health Questionnaire 15 (PHQ-15)

In OFP and TMD sufferers somatization is prevalent in primary care [52]. Importantly, the PHQ-15 items were not used as a measure of somatization severity according to its original publication [30]. Rather than using the PHQ-15 as a validated questionnaire, its item content was included in the checklist to coarsely assess the burden of physical/bodily symptoms beyond OFP AND TMD (see Table 3). E.g., content of PHQ-15 items 1 (stomach pain), 4 (menstrual cramps or other problems with your periods), 6 (chest pain), and 11 (pain during sexual intercourse or other sexual problems) were slightly modified to focus on pain and grouped into checklist item 13. Content of PHQ-15 items 7 (dizziness), 8 (fainting spells), 9 (feeling your heart pound or race), 10 (shortness of breath), and 13 (nausea, indigestion) were grouped into WISE checklist item 18, to screen for symptoms associated with autonomic dysfunction. PHQ-15 item “trouble sleeping” was replaced by content of item 3 of PHQ-9 item “trouble falling or staying asleep, or sleeping too much.” Because of the analogous scoring, it was placed after the PHQ-4 section in the checklist. We considered the PHQ-9 item content more appropriate since it covers a broader spectrum of sleep problems.

**Table 3:** Publicly available questionnaires used in the construction of the WISE for OFP and TMD

Domain	Questionnaire (Abbreviation)	Type of scales, number of items	Range per item	Maximum score
				Possible cut-off values for further evaluation
Stress	Patient Health Questionnaire Stress (PHQ-S) [24]	ordinal 10	0–2	20 10–14: moderate >14: severe
Anxiety	General Anxiety Questionnaire 7 (GAD-7) [31]	ordinal 7	0–3	21 7–12 [31, 33, 73–75]
Depression	Patient Health Questionnaire 9 (PHQ-9) [32]	ordinal 9	0–3	27 8–11 [31, 33]
Pain related disability	Graded Chronic Pain Scale (for orofacial pain/body pain) (GCPS) [34]	numeric 7	0–10	6 3 [34]
Pain catastrophizing	Pain Catastrophizing Scale (PCS) [36]	ordinal 4	0–4	52 30 [36]
Illness perception	Brief Illness Perception Questionnaire (B-IPQ) [41]	numeric 8	0–10	80 n/a
Injustice experience	Injustice Experience Questionnaire (IEQ) [43]	ordinal 12	0–4	48 30 [43]
Dysmorphic concern	Dysmorphic concern questionnaire (DCQ) [44]	ordinal 7	0–3	21 9 [44]
TMD Symptoms	DC-TMD Symptom Checklist [76] (DC-TMD-SQ)	Checklist 14	0–1	n/a
Jaw function	Modified Jaw Function Questionnaire (JFQ) [49, 50]	Checklist	0–1	n/a
		10		
		nominal 2	0–10	n/a
Tinnitus	Tinnitus Handicap Inventory 12 (THI-12) [46]	ordinal 12	0–2	24 10 [46]
Headache	ID-migraine screener [48]	Checklist 3	0–1	3 2 [48]
Sleep	Insomnia Severity Index (ISI) [40]	ordinal 7	0–4	28 14 [37–39]
Somatosensory dysfunction	Somatosensory Symptom Checklist (SSC) [51]	Checklist	0–7	n/a

#### PHQ-Stress

The content of PHQ-Stress items were transformed into checklist items, to screen for psychosocial stressors. Content of PHQ-Stress item 2 (your weight or how you look) was covered by checklist items 8 and 16. Content of PHQ-Stress item 3 (little or no sexual desire or pleasure during sex) was covered by checklist item 13. Content of PHQ-Stress items 5 (stress of taking care of children, parents, or other family members), 6 (stress at work outside of the home or at school), and 7 (financial problems or worries) were grouped into checklist item 19. Content of PHQ-Stress items 4 (interpersonal conflicts) and 8 (lack of support/loneliness) were grouped into checklist item 20. Content of PHQ-Stress items 9 (something bad that happened recently) and 10 (thinking or dreaming about something terrible that happened to you in the past) were grouped into checklist item 22. Content of items 2 and 3 were not integrated into checklist because their content was already covered by other checklist items.

#### Patient Health Questionnaire 4 (PHQ-4)

The PHQ-4 was implemented in its original version, except that the recall period was adapted to “over the last 30 days,” for covering the same time interval in all WISE assessments. The sleep item of PHQ-9 was added below the PHQ-4 due to the analogous scoring.

#### Optional checklist items

Patients experiencing ongoing pain associated with invasive procedures may experience feelings of injustice. Additionally, some patients may feel that they are a burden to others. For screening purposes, these aspects were captured by the two items listed in Table 4.

**Table 4:** Checklist items capturing aspects of injustice experience and being a burden to others

Checklist item (continued)	Source	Threshold	In-depth assessment
28) Did you experience injustice concerning your chief complaints (e.g., misinformation, mistreatment, undue expense etc.)?	none	yes	IEQ
29) Are you concerned about being a burden to others?	none	yes	B-IPQ

Depending on the needs of a given clinic or research focus, additional items can be included. In addition to the items listed in Table 4, further examples are listed in Table 1 (items 10, 11, 17 and 21).

### Pain related in-depth questions

Upon exceeding a predefined checklist threshold value, the following items were presented.

#### Pain severity and location

Various tools measure pain severity. We chose the NIH Toolbox as it is widely accepted. It uses an 11-point intensity rating scale with anchors 0 (“no pain”) and 10 (“worst imaginable pain”) [53]. For consistency, we implemented these anchors in the entire WISE.

**Fig. 2:**
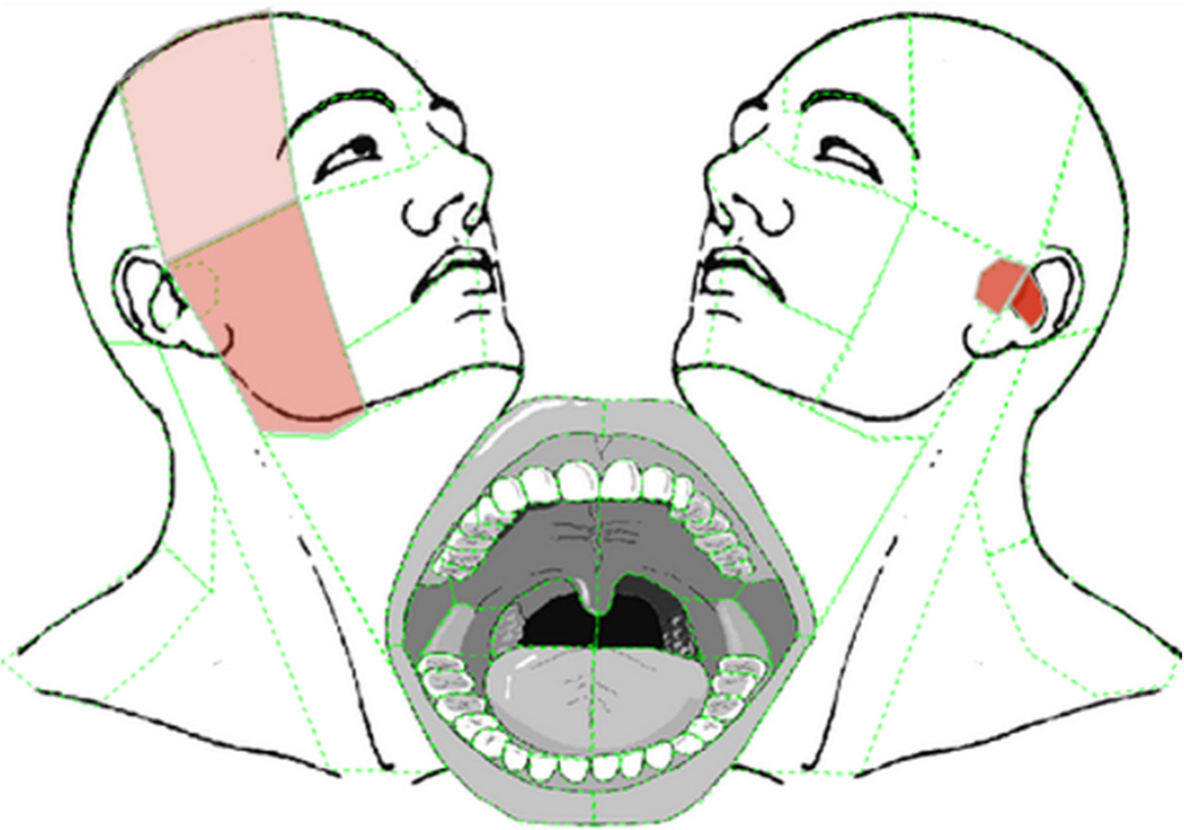
Pain drawing. The predefined areas are marked with *green dotted lines*. Pain intensity of selected regions is represented by gradients of *red*. Darker *red* indicates more intense pain

For different regions of the head, face, and mouth, pain intensity during the last 4 weeks was assessed using a pain-drawing tool. On an image of the head and oral cavity, predefined regions relating to underlying anatomical structures could be selected with a mouse-click (e.g., masticatory muscles, teeth; Fig. 2). IM was used to make these areas selectable and to color selected regions, with darker red denoting greater pain intensity. Patients first click on the area of the most burdensome pain, which revealed a dialog querying the most frequent pain intensity at rest and maximum pain intensity on jaw movement.

#### Pain quality

Pain quality was characterized by the descriptors (Schmerzbeschreibungsliste; SBL) of the German pain questionnaire (Deutscher Schmerzfragebogen; GPQ). The list was supplemented with adjectives that capture distinct OFP perceptions [54, 55]. Patients were requested to choose which of 15 pain descriptors described their typical current pain and pain at illness onset. The list included nine sensory pain adjectives (“dull-pressing,” “pulling,” “stinging,” “pulsating-throbbing,” “burning-hot,” “pins and needles,” “shooting-electric,” “tingling,” “numb”) and six affective pain adjectives (“dreadful-horrible,” “miserable-atrocious,” “exhausting,” “grueling,” “agonizing,” “frightening”). The option “other pain quality” allowed patients to add descriptors that were otherwise not covered.

#### Pain duration

Because the time value that defines chronic pain varies from 3 to 6 months, we added an interval ranging from 3 to 6 months to the classification used in the GPQ [54, 56]. We included the following time intervals: *“up to 3 months*,” *“more than 3 up to 6 months*,” *“more than 6 months up to 2 years*,” *“more than 2 years up to 5 years*,” and *“more than 5 years*.”

#### Time pattern

**Fig. 3:**
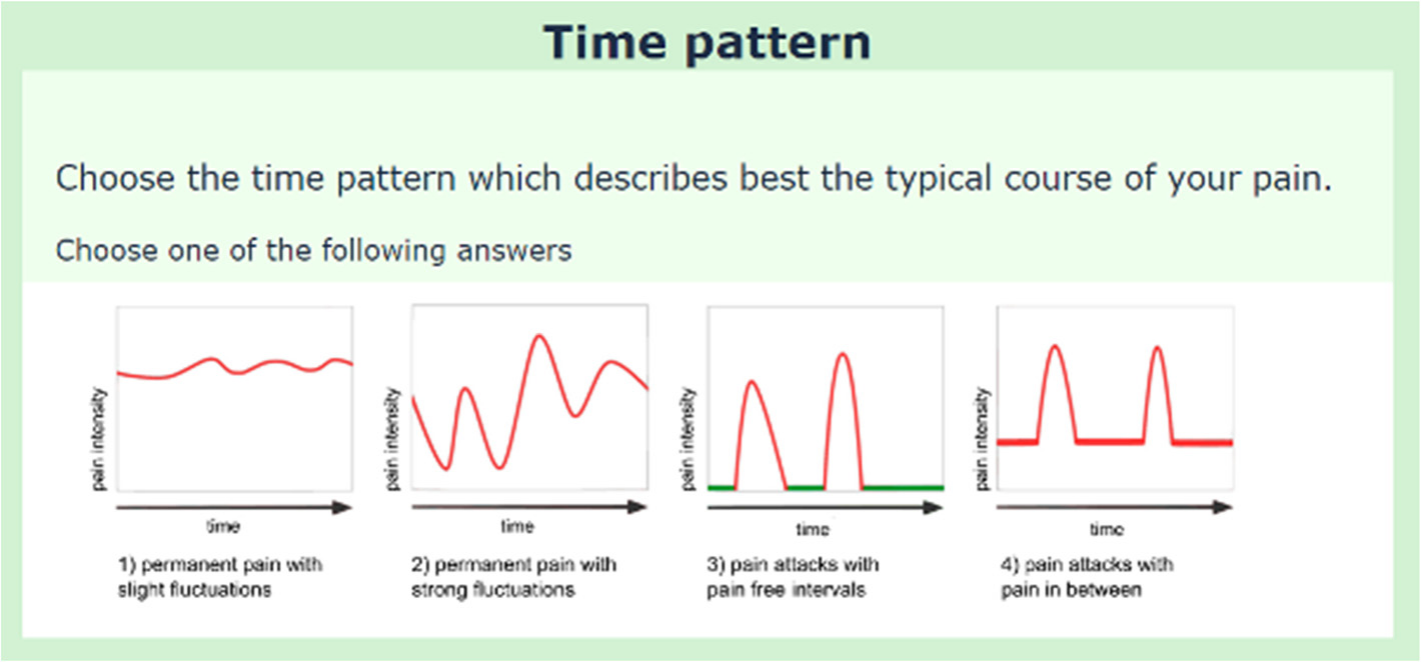
Time pattern of pain. Patients can select one of four different patterns

For the most burdensome pain, time variation was assessed by one of four different patterns, according to the GPQ [54] (Fig. 3). Patients chose the time pattern that best matched their typical pain course. If pattern 2, 3, or 4 was chosen, the following item was presented: “Please indicate the intensity of the most frequent maximum pain during the last 4 weeks.” Choosing pattern 3 or 4 was followed by the items: “How often do these attacks typically occur while awake? (number of attacks per 24 h)”, “How often do these attacks typically occur while sleeping? (number of attacks per 24 h)”, “Which is the typical duration of your attacks?” and “The attacks are triggered by …?”.

#### Diurnal pain course

**Fig. 4:**
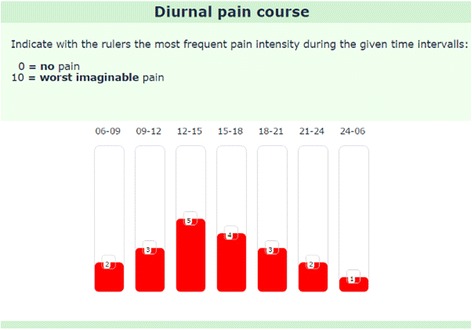
Example of a possible diurnal pain course. Each bar represents a 3-h time period during the day and a 6-hour period at night. Most frequent pain intensity from 0 to 10 is indicated by moving the bars accordingly

For the most burdensome pain, seven sliders were used to represent the most frequently experienced intensity during 3-hour intervals throughout the day and one 6-h interval at night (Fig. 4). Because maximum pain severity was already captured in section Pain severity and location (Fig. 2), the “most frequent” pain was considered more relevant in this context. The ill-defined term “average” pain intensity was intentionally avoided.

#### Onset of pain

Possible reasons for the onset of pain were captured by a single-choice checklist with the options a) “gradual,” b) “sudden,” c) “by event (accident, physical/emotional stress, dental/medical treatment, operation, illness),” and d) “other.” If applicable, further sub-items explored the date of first memorable occurrence and the exact nature of events.

### Single case summary report

**Fig. 5:**
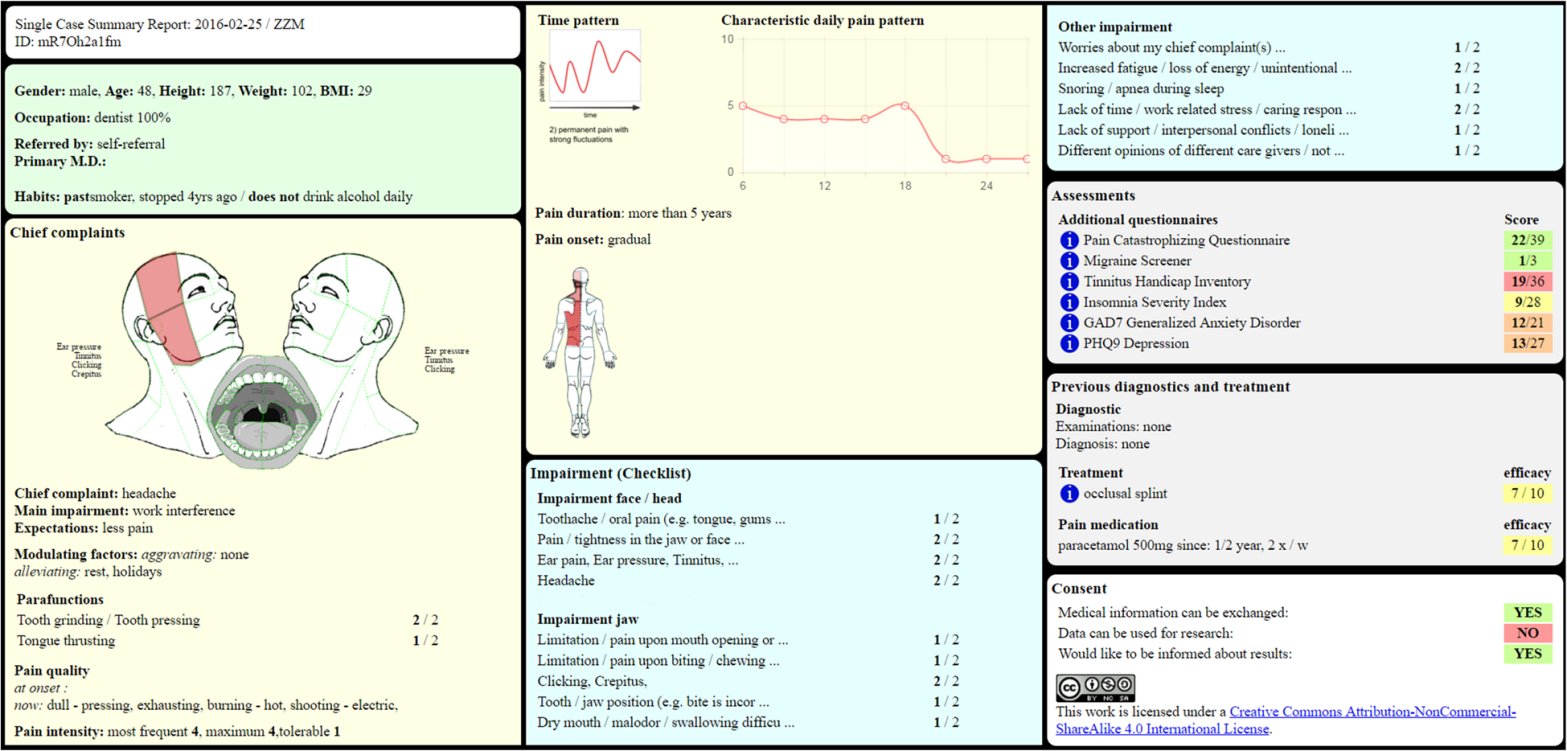
Example of a single case summary report

**Fig. 6:**
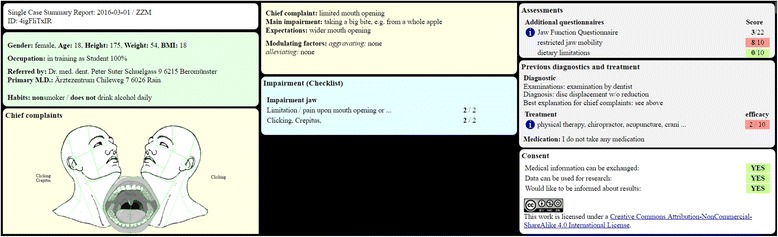
Example of a single case summary report

Single case summary reports varied in length, depending on case complexity. We present two examples of the computer-generated data assembly in Figs. 5 and 6. Note that the report is interactive. Detailed results of in-depth questionnaires can be displayed by moving the cursor over selected areas of images or over the ⓘ button.

### Language options

Multilingual surveys are optional in LS.

## Discussion

Francis W. Peabody opined that “the secret of the care of the patient is in caring for the patient” [57]. Today, health IT offers new ways of identifying patient needs by comprehensively assessing the varied biopsychosocial factors that influence the experience of pain and other symptoms. From a conceptual point of view, the WISE prioritizes the subjective symptom burden. Many symptoms in the orofacial regions differentially burden individuals such as jaw joint noises, feeling of tension in the masticatory muscles, C-shaped jaw deviation, etc. Whereas they are barely bothersome to some people, they can be majorly burdening to others. This experiential disparity is often linked to psychological comorbidities unidentified in the primary (dental) care setting. In many everyday clinical situations, patient management will be symptom oriented and the indication for interdisciplinary (e.g. psychological) evaluation will depend mostly on symptom burden. Commonly the therapeutic strategy for patients suffering from musculoskeletal, neuropathic or idiopathic pain in the orofacial region is targeted towards symptom relieve rather than elimination of etiologic factors as these are often unknown. Thus, the WISE supports clinical decision making in clinical practice aiming at identifying patients’ needs (based on symptom burden) for optimal care, possibly including an interdisciplinary approach.

The scoring of WISE checklist items focuses on symptom-related burden, per J. D. Loeser: “It is suffering, not pain, that brings patients into doctor’s offices in hopes of finding relief” [58]. Besides diagnostic performance, questionnaires are ideally brief, self-administered, multipurpose, free, and easy to score. All these features were considered in generating the WISE.

We described in detail the design, construction, and technical implementation of web-based questionnaire for assessing patients with OFP and TMD. It was designed to be modular, flexible, extensible, and to include drawing options as well as instructional videos. The use of open-source software tools and freely available questionnaires prevented copyright infringements. Secure data storage limits access strictly to those who use the WISE for collecting, storing, and evaluating their own data. Although WISE and the US-based CHOIR have similarities, no available manuscript reports on the structure and composition of its OFP andTMD questions, its algorithm, or its scoring system [18].

*For patients*, a major advantage is that the WISE is available independent of location; therefore respondents can provide information without time pressure via any available computer, on all possible platforms, using any standard browser, and in different languages. The WISE’s modular design is highly patient-centered as it enables personalized assessment of biopsychosocial burden. The option to stop and restart at any point in time diminishes the cognitive burden and respondents can easily review and revise entered data before submission. Respondents are not dependent upon planned, prearranged clinical appointments.

*For care providers* and administrative personnel, the WISE is easy to administer and electronic data are stored securely. The system can even be used by clinicians who lack electronic health records. The tool’s modular structure enables organization of relevant information prior to patient appointments, thus facilitating time and personnel management. Namely, information on somatic and psychosocial burdens may clarify the need for interdisciplinary consultation to identify an appropriate expert. The single case summary report of WISE enables a focused clinical evaluation that prioritizes the most burdensome complaints. This likely facilitates caregiver-patient interactions as from the outset the patient feels understood regarding his/her chief complaint.

*For educators*, the WISE can assist interprofessional education (IPE) in this field [59]. IPE aims to share skills and knowledge among health care disciplines. WISE case reports in a teaching environment can illustrate to students benefits to patients of working within interprofessional teams [60]. Implementation of such educational models has been recommended by the Institute of Medicine of the US National Academies [61].

*Clinical researchers* benefit from the WISE by having available standardized data sources. A database is generated without the need for cumbersome transcription of paper-based tools. Central data storage allow for prospective data collection and aggregation from different centers. Novel study designs can be initiated that overcome limitations of conventional randomized controlled clinical trials, e.g., comparative (real world) effectiveness studies [62–65]. Biopsychosocial phenotypes identified through research can be used in clinical practice for more refined screening and more tailored management [66].

*For health insurance carriers and other third-party financing agents,* personalized health contributes directly to cost savings, but also indirectly by reducing the risk of chronicity.

Finally, wide implementation of the WISE will aid to adequately and comprehensibly incorporate psychosocial entities in classification systems rooted in an ontological framework based on analysis of symptom clusters [67–72].

The WISE is extensible: the composition of symptom burden checklist items and/or case finding instruments can be modified, depending on the needs of a given clinic, e.g., for more detailed exploration of obstructive sleep apnea, halitosis, xerostomia, dysphagia, etc.

### Limitations

There are limitations of web-based instruments: Compared to paper-based versions, completing electronic questionnaires is more time consuming and some people may require assistance. With increasing familiarity with electronic devices, this problem will likely diminish. In the context of clinical trials, most patients prefer electronic data collection methods [16]. Whether the web-based administration significantly decreases patient burden and increases compliance will require further evaluation.

In this paper, the WISE symptom domains were selected for a single interdisciplinary OFP and TMD care team in Zurich. Future studies are warranted to clarify the validity of the chosen symptom burden checklist structure. We opted for the above mentioned case-finding questionnaires based on the following priority sequence: free availability > brevity > robust psychometric properties in the primary care setting. This choice was an arbitrary decision by the authors and not based on an international expert panel recommendation.

The WISE was designed to assist clinical decision making. Whether it also has psychometric validity requires further testing. Also, determination of the optimal thresholds for opening a case-finding instrument needs further clarification. Further, the utility of the instrument to detect change over time for determining treatment effects will need to be clarified. Our planned future research will focus on these issues.

The current version of the WISE uses seven vertical rulers for obtaining a general impression of the diurnal pain course. This is a common limitation of electronic data gathering compared to a pencil-paper approach where pain courses can easily be drawn. Also fluctuating pain patterns cannot be captured by this tool. Still, we estimate that the combination of the diurnal pain course combined with the general pain pattern will offer an initial impression of most clinically relevant pain patterns. However, this assumption will require future scientific assessment.

The WISE for OFP and TMD is only one part of an integral patient evaluation that also includes an interview, physical examination, imaging, laboratory tests, other expert evaluations, etc. How all these additional data can be integrated in a comprehensive patient database remains to be explored.

## Conclusions

The WISE is a novel web-based tool that assists clinicians in clarifying case complexity and referral need, based on symptom burden and response –tailored case finding. It provides single-case summary reports from a biopsychosocial perspective and includes graphical symptom maps. Secure, centrally stored data collection of anonymous data is possible. The tool enables personalized medicine, facilitates interprofessional education and collaboration, and allows for multicenter patient-reported outcomes research.

## Summary

We presented the design, construction, and technical implementation of a web-based instrument for interdisciplinary evaluation of symptom burden, illustrated for OFP and TMD.

## Abbreviations

B-IPQ: Brief illness perception questionnaire
CHOIR: Collaborative health outcomes information registry
DCQ: Dysmorphic concern questionnaire
DC-TMD: Diagnostic criteria for temporomandibular disorders
DC-TMD-SQ: DC-TMD Symptom Questionnaire
GAD-2: Short form of the General Anxiety Disorder Questionnaire 7
GAD-7: General anxiety disorder screener
GCPS: Graded chronic pain scale v2
GPQ: Deutscher Schmerzfragebogen
ID: Identification number
ID-Migraine™: Identification of Migraine
IEQ: Injustice experience questionnaire
IM: Image mapster
ISI: Insomnia severity index
IT: Information technology
JFQ: Jaw function questionnaire
LS: LimeSurvey
OFP: Orofacial pain
PCS: Pain catastrophizing scale
PHQ: Patient health questionnaire
PHQ-15: Patient health questionnaire 15
PHQ-2: Short form of the Patient Health Questionnaire 9
PHQ-4: Patient health questionnaire 4
PHQ-9: Patient health questionnaire 9
PRIME-MD: Primary care evaluation of mental disorders
RCD/TMD: Research diagnostic criteria for temporomandibular disorders
SBL: Schmerzbeschreibungsliste
SDATA: Survey data
SSC: Somatosensory symptom checklist
THI-12: Tinnitus handicap inventory 12
TMD: Temporomandibular disorder
UZH: University of Zurich
WISE: Web-based interdisciplinary symptom evaluation
